# Stochasticity in action potential backpropagation: consequences for neuronal computation

**DOI:** 10.3389/fncel.2026.1803262

**Published:** 2026-03-23

**Authors:** Srdjan D. Antic, Katarina D. Milicevic, William W. Lytton

**Affiliations:** 1 Department of Neuroscience, UConn Health, School of Medicine, Institute for Systems Genomics, Farmington, CT, United States; 2 Institute for the Brain and Cognitive Sciences (IBACS), University of Connecticut, Storrs, CT, United States; 3 Department of Physiology and Pharmacology, SUNY Downstate Health Sciences University, Brooklyn, NY, United States

**Keywords:** dendritic integration, network computation, pyramidal neurons, spike-timing-dependent plasticity, stochastic neural signaling

## Abstract

In cortical and hippocampal pyramidal neurons, backpropagating action potentials (bAPs) play a central role in dendritic signaling, synaptic integration, and spike-timing-dependent plasticity (STDP). In most experimental and theoretical frameworks, bAPs are implicitly treated as reliable signals that faithfully inform dendritic synapses of somatic spiking. Here, we review experimental evidence demonstrating that this assumption is often violated. In large portions of the pyramidal neuron dendritic tree, particularly in distal apical branches and apical tuft dendrites, bAP amplitude exhibits pronounced spatial and temporal variability, including: (i) activity-dependent attenuation, (ii) frequency-dependent amplification, (iii) branch-specific propagation failures, and (iv) trial-to-trial stochastic AP flickering. We summarize five experimentally documented forms of bAP variability and discuss how stochastic backpropagation may shape synaptic plasticity in computational neuroscience, especially STDP, by introducing probabilistic gates that limit the coincidence of: (i) dendritic depolarization (bAP) and (ii) synaptic input (EPSP). Finally, we consider broader implications of the AP flickering in dendrites for cortical information processing, including redundancy, averaging, evidence accumulation, and error-correcting strategies in cortical circuits.

## 1 Introduction

Nerve impulses (action potentials; APs) are fundamental to neuron-to-neuron communication. Through generation of APs, telencephalic pyramidal cells (cortical and hippocampal) convey output to diverse postsynaptic targets including hippocampus, ipsilateral and contralateral cortex, thalamus, pons, superior colliculus, spinal cord, and other regions. Two foundational themes in systems neuroscience: (a) the formation of neural ensembles (Buzsaki, 2010), and (b) the emergence of brain oscillations (Traub et al., 1996), critically depend on AP generation and propagation. In pyramidal neurons, synaptic inputs arrive predominantly on dendrites and are integrated across dendrites and soma; nevertheless, the AP typically initiates at the axon initial segment (AIS), where a high density of voltage-gated sodium channels (Kole et al., 2008) and a small diameter (Fekete et al., 2021) lower spike threshold. From the AIS, the AP simultaneously propagates in two directions: Orthograde, from AIS to distal axon (*Forward AP propagation* (Popovic et al., 2011)) and Retrograde, from AIS to distal dendrites (*AP Backpropagation* (Stuart et al., 1997)). The primary physiological consequence of the backpropagating AP (bAP) is dendritic depolarization, which can engage synaptic and intrinsic conductances relevant to: (i) plasticity, and (ii) dendritic output. Briefly, dendritic depolarization serves two key functions:

First, it can facilitate activation of dendritic NMDA receptors when glutamate is present, either during ongoing synaptic transmission (Schiller et al., 1998), or when glutamate persists following strong synaptic activity (Santos et al., 2012).

Second, dendritic depolarization can recruit voltage-gated calcium channels (VGCCs), increasing local Ca^2+^ entry (Jaffe et al., 1992) and, in some contexts, promoting release of retrograde messengers (e.g., endocannabinoids). In this mode, dendrites can signal back to presynaptic terminals and nearby cells, effectively bypassing the traditional neuronal reliance on axonal signaling (Fortin et al., 2004).

Because bAP-driven dendritic depolarization can activate both NMDA receptors (Schiller et al., 1998) and VGCCs (Jaffe et al., 1992), long-term information storage via synaptic plasticity depends, in many synapses, on effective AP backpropagation. By varying the interval between a dendritic excitatory postsynaptic potential (EPSP) and the arrival of a bAP from the soma, synapses can undergo long-term potentiation (LTP), or long-term depression (LTD) (Magee and Johnston, 1997; Markram et al., 1997). The cellular process that takes into account the timing of an AP in relation to the timing of the synaptic input, to determine the direction of the synaptic modulation (increase or decrease) is called “spike timing dependent plasticity”, STDP (Song et al., 2000). STDP is widely used in computational models of learning and cognition (Masquelier et al., 2008; Bush et al., 2010; Clopath et al., 2010; Pecevski and Maass, 2016) However, most such models implicitly assume that bAPs are reliable signals, and therefore overlook empirically observed stochasticity in bAP invasion and amplitude.

**Figure 1:**
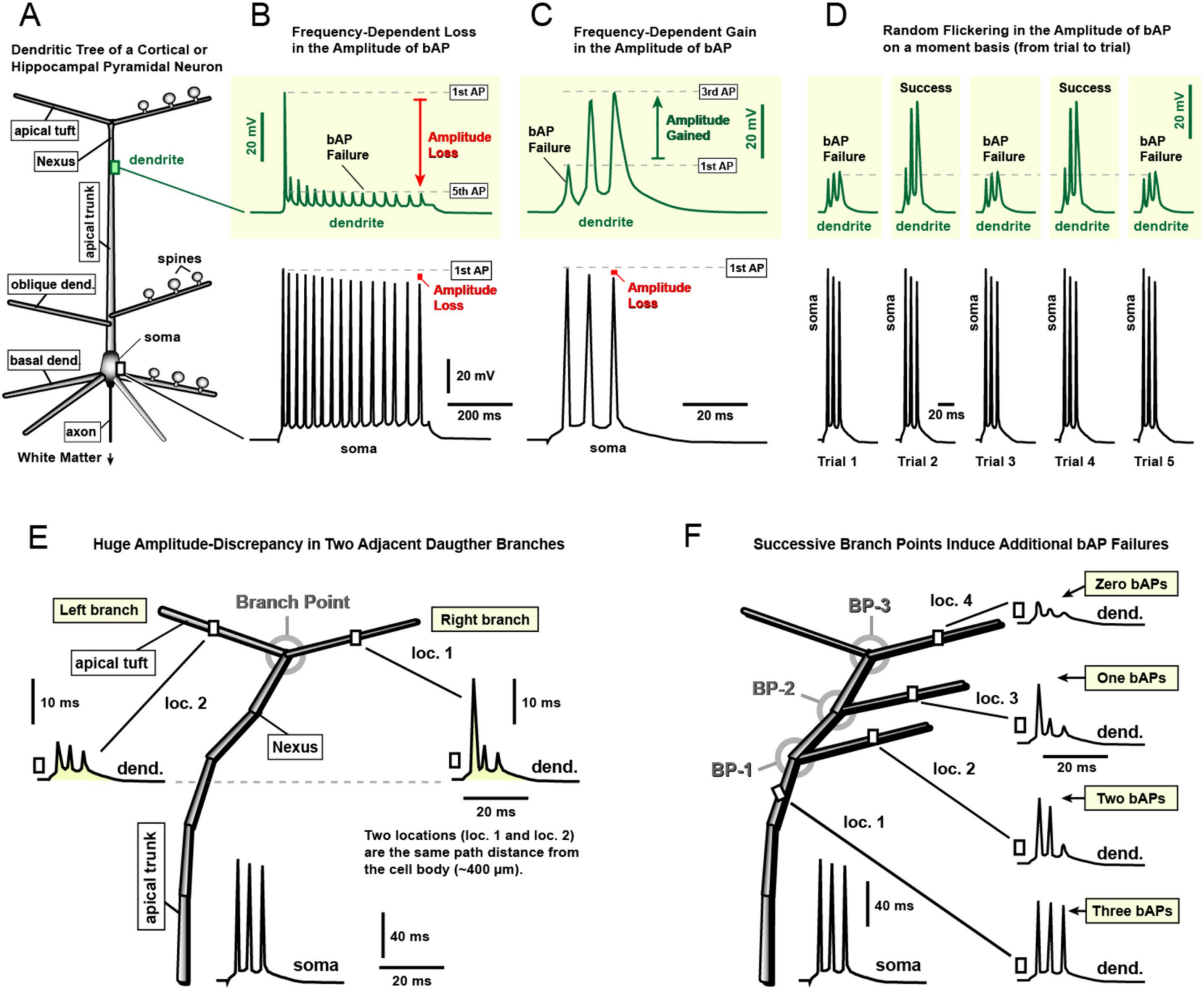
Backpropagating action potentials (bAPs) are unreliable electrical signals. **(A)** Schematic of a pyramidal neuron illustrating basal, apical, oblique, and tuft dendritic branches. Dendritic spines, which mediate synaptic transmission and synaptic plasticity underlying learning and memory, are shown schematically. Recording sites at the soma and distal apical dendrite are indicated by colored rectangles. **(B)** Simultaneous recordings from the soma (black) and distal apical dendrite (green) reveal progressive attenuation of bAPs occurring later in an action potential (AP) train. Amplitude reductions relative to the first AP are indicated by red symbols. This panel is based on experimental findings reported in Spruston et al. (1995) their Figure 2, Magee and Johnston (1997) their Figure 2A, Williams and Stuart (2000) their Figure 1B, Gulledge and Stuart (2003) their Figure 1A, and Larkum et al. (2001) their Figure 8B. **(C)** Same neuron type (pyramidal) and same dendritic compartment (distal apical) as in panel *B.* A higher somatic firing frequency leads to activity-dependent enhancement of bAP amplitude. The first AP fails to invade the dendrite, whereas the subsequent two APs successfully propagate. The amplitude increase relative to the first AP is indicated by the green arrow. This effect is based on: Larkum et al. (1999) their Figure 3C, Williams and Stuart (2000) their Figure 5A, and Short et al. (2017) their Figure 9A2. **(D)** Repeated recordings obtained every 4 min (trials 1–5) from the same distal dendritic segment of the same neuron reveal pronounced trial-to-trial variability (“flickering”) in bAP amplitude. In most trials (Trials 1, 3, and 5), bAPs failed to invade the dendritic site, whereas in some trials (Trials 2 and 4) they stochastically successfully invaded the distal dendritic segment with sufficient amplitude to activate local VGCCs. This result is based on: Short et al. (2017) their Figures 3, 4. **(E)** Schematic of the apical tuft of a pyramidal neuron. Somatic stimulation evokes a train of three APs (soma). Two dendritic branches located at the same path distance from the soma experience markedly different membrane voltages at the same time (compare loc. 1 vs. loc. 2). This result is based on: Spruston et al. (1995) their Figure 4A, and Zhou et al. (2015) their Figure 6AB. **(F)** Somatic stimulation evokes a train of three APs (soma), while membrane voltage is recorded at four locations within the apical tuft (loc. 1–4). Despite identical somatic input, these locations experience substantially different voltages at the same moment. Branch points (BP-1 to BP-4) are a major determinant of this variability: passage of the AP triplet through successive branch points increases the probability of bAP propagation failure, reducing the likelihood of invasion into each daughter branch. Based on: Spruston et al. (1995) their Figure 5, and Zhou et al. (2015) their Figure 7. References used for designing this figure: Spruston et al. (1995), Magee and Johnston (1997), Larkum et al. (1999), Williams and Stuart (2000), Larkum et al. (2001), Gulledge and Stuart (2003), Zhou et al. (2015), and Short et al. (2017).

**Figure 2:**
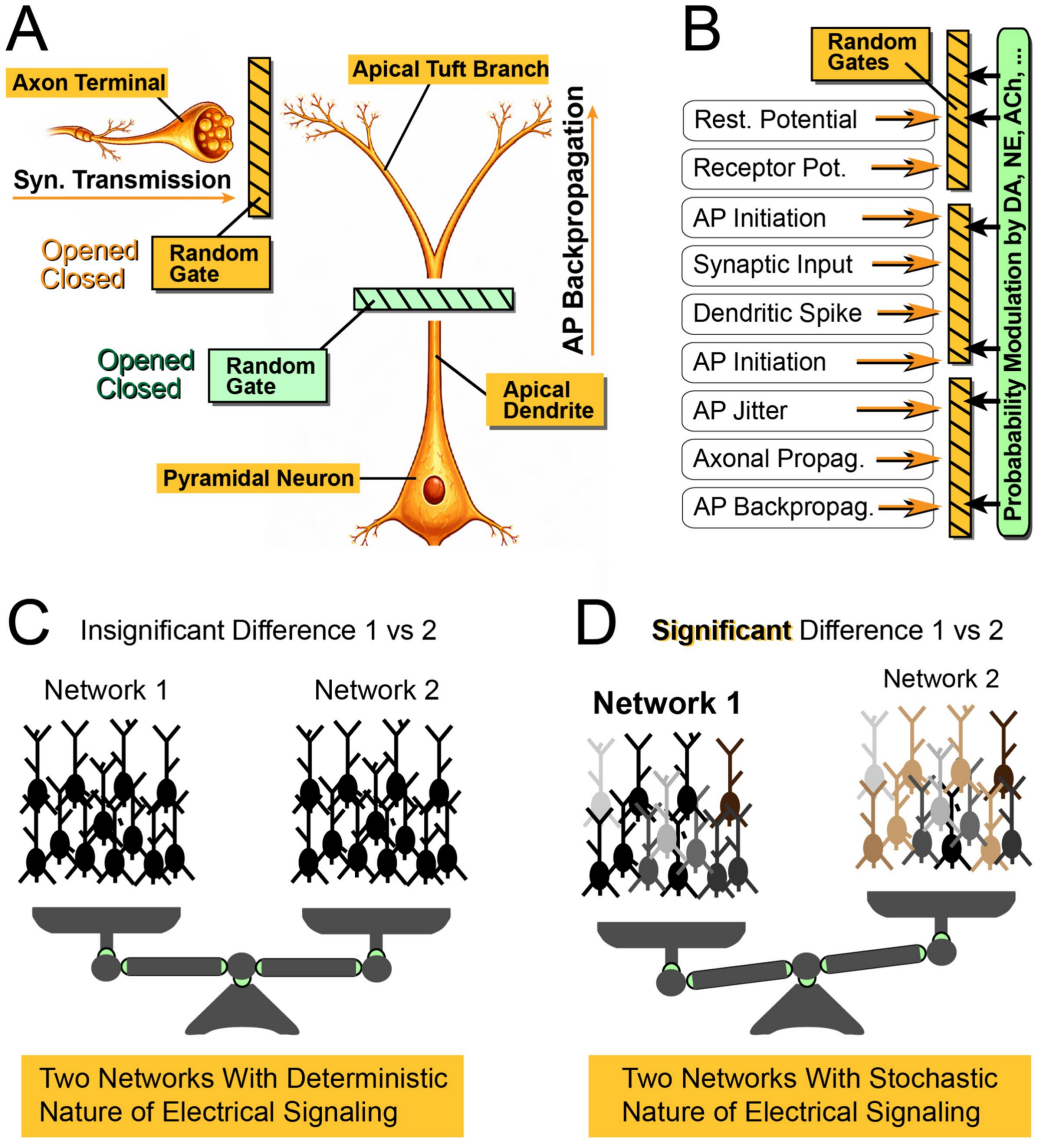
Probability gates in cortical information processing. **(A)** Schematic of a pyramidal neuron receiving a single excitatory synaptic input onto an apical tuft branch (Syn. Transmission). Between the presynaptic axon terminal (Axon Terminal) and the postsynaptic dendritic compartment (Apical Tuft Branch), a functional gate is interposed that stochastically permits or blocks the transmission of EPSPs (Random Gate). A second gate along the apical dendrite regulates the invasion of apical tuft branches by backpropagating action potentials (bAPs), such that the probability of bAP invasion is less than unity. **(B)** Each mode of electrical signaling is controlled by its own probabilistic gate, characterized by a specific probability of occupying an open or closed state. These probabilities can be dynamically modulated by biological processes (e.g., neuromodulation), thereby increasing or decreasing the likelihood that a given gate is open. Briefly, an open-state probability of each random gate is modifiable by a biological process dubbed “Probability Modulation.” **(C)** Two competing networks operating with deterministic signaling (100% success rate). Each presynaptic action potential reliably evokes a postsynaptic EPSP (gate is always open), and each somatic action potential fully invades the apical tuft (gate is always open). Network outputs correspond to the weighted average activity across neurons, with darker shading indicating stronger activity. Black color indicates a very strong activity of the nerve cell. After several computational iterations (information-integration periods), the two networks produce similar output intensities due to the absence of probabilistic modulations. **(D)** When electrical signaling is constrained by probabilistic gates (Random Gate), neuronal activity becomes more diverse. In this example, Network 1 contains a slightly greater number of active (dark) neurons than Network 2, yielding a clearer separation in network output. As a result, the output of Network 1 is preferentially selected to guide behavior.

In this review, we first describe five experimentally supported conditions in which AP backpropagation is not robust but instead exhibits probabilistic outcomes (Figure 1). We then speculate on how variability in bAP “outcomes” could influence synaptic plasticity and information processing in cortical circuits (Figure 2). Throughout, we use the term “pyramidal neuron” to refer primarily to: (i) neocortical layer 5 pyramidal neurons, and (ii) hippocampal CA1 pyramidal neurons.

## 2 Electrical signal that constantly varies

In experimental studies of STDP (Bi and Poo, 1998), and in many STDP-based computational theories (Song et al., 2000), bAP are commonly treated as reliable: each somatic AP is assumed to deliver a stereotyped depolarization to the dendritic synapse. Here, we argue that this assumption is often incorrect. We summarize evidence that, across large portions of the pyramidal dendritic tree, particularly in branching regions and in distal segments of the dendritic tree, the voltage transient associated with a bAP can vary substantially from spike to spike, and from trial to trial, including partial invasions and complete propagation failures. In some distal dendritic branches, the voltage signal from backpropagating AP fluctuates from trial to trial, from moment to moment, in an unpredictable manner, stochastically (Short et al., 2017).

Here we define “AP backpropagation failure” as the loss of regenerative spike generation in a local dendritic segment, resulting in a strongly attenuated voltage signal. In dendritic regions where the action potential fails to propagate regeneratively, the membrane depolarization is not zero. Instead, a fraction of the voltage spreads electrotonically from adjacent proximal segments where the spike remains regenerative. Consequently, the distal dendritic segment experiences a passive depolarization that reflects attenuated propagation of the back-propagating spike. In this sense, bAP failure differs conceptually from synaptic failure: during synaptic failure, no postsynaptic depolarization is generated, whereas during bAP failure a subthreshold depolarization may still be present due to passive voltage spread. Examples of failed APs are shown in three panels of Figures 1B–D and labeled as “*bAP Failure*.”

### 2.1 bAP amplitude variations based on the preceding activity—amplitude loss

If a synapse is located on the apical dendrite (Figure 1A, apical trunk, oblique branches, or tuft), bAP amplitude depends strongly on recent spiking history. During repetitive firing (∼10–50 Hz), somatic APs show only modest amplitude attenuation (Figure 1B, soma), whereas dendritic APs often exhibit pronounced, voltage-dependent amplitude loss (Figure 1B, dendrite). At a fixed dendritic location, bAP amplitude can range from near-full invasion (peaks 60 mV above the dendritic resting potential) to apparent failures (<20 mV) within the same spike train. For example, ~350 μm from the soma, the first AP may depolarize the dendrite by ~60 mV, while later spikes in the train may fail to reach 20 mV (Spruston et al., 1995; Larkum et al., 2001). Therefore, the first spike successfully invaded the dendritic segment (~60 mV peak amplitude), whereas the later spike, with a peak amplitude of only ~20 mV, represents a genuine bAP failure (see definition in the previous paragraph).

Activity-dependent attenuation of bAP amplitude has been reported in multiple studies of apical trunk excitability (Callaway and Ross, 1995; Spruston et al., 1995; Magee and Johnston, 1997; Williams and Stuart, 2000; Larkum et al., 2001; Gulledge and Stuart, 2003), and is mediated, in part, by cumulative Na^+^ channel inactivation that can begin after a single spike and progress during subsequent spikes (Migliore, 1996; Colbert et al., 1997; Jung et al., 1997). Consequently, the effective coupling between a dendritic input (EPSP) and a somatic spike (AP) depends on the immediate spiking history preceding synaptic input.

### 2.2 bAP amplitude variations based on the preceding activity—amplitude gain

In the same cell types (cortical and hippocampal pyramidal neurons) and within the same dendritic compartment (the apical dendrite), increasing somatic firing frequency to ~90 Hz can produce the opposite outcome: an activity-dependent gain in bAP amplitude in distal dendrites during a spike train. Rather than undergoing the progressive attenuation, as observed at lower firing rates (Figure 1B), bAPs under high-frequency conditions can exhibit increasing voltage amplitudes in distal dendritic segments (Figure 1C). In this regime, later spikes invade the distal dendrite more effectively, resulting in progressively larger dendritic depolarizations. This result was not inferred from calcium imaging; it is based on dendritic measurements of voltage waveforms (Larkum et al., 1999; Williams and Stuart, 2000; Short et al., 2017).

A critical prerequisite for this facilitation is that the first AP in the train fails to fully invade the distal dendrite (peak amplitude <20 mV; Figure 1C, 1st AP). Computational models indicate that such bAP failures occur preferentially in branches with a high local density of A-type K^+^ channels (Short et al., 2017, their Figure 8) (Zhou et al., 2015; Short et al., 2017). Consistent with this mechanism, pharmacological blockade of A-type K^+^ channels markedly increases bAP amplitudes in distal dendritic branches and beyond the apical dendrite branch points (Gasparini et al., 2007; Zhou et al., 2015; Short et al., 2017). In untreated pyramidal neurons, although the initial bAP may fail to propagate fully into the distal dendrite (Figure 1C, 1st AP), the resulting depolarization is sufficient to partially inactivate dendritic A-type K^+^ channels. Consequently, subsequent spikes encounter reduced outward K^+^ current and propagate more robustly into the apical tuft (Short et al., 2017; Park et al., 2025). Note that the second and third APs show progressively larger voltage responses (Figure 1C, dendrite). In some cases, depolarizations from the first and second bAP contribute to cumulative A-type K^+^ channel inactivation, allowing the third bAP to reach the largest peak amplitude and exhibit the slowest repolarization (Figure 1C, green trace), consistent with reduced voltage-gated K^+^ conductance. The role of dendritic A-type K^+^ channels in regulating bAP propagation has been extensively characterized by the Johnston laboratory (Hoffman et al., 1997; Migliore et al., 1999).

The coexistence of these two opposing regimes, amplitude-loss (Figure 1B, red arrow) and amplitude-gain (Figure 1C, green arrow), within the same pyramidal neuron type and dendritic class underscores the strong state dependence of bAP efficacy. In the amplitude-loss regime (Figure 1B), dendritic depolarization is weakest when synaptic input arrives late in the somatic spike train. Conversely, in the amplitude-gain regime (Figure 1C), dendritic depolarization is weakest when synaptic input coincides with early spikes. Because bAP-induced dendritic depolarization is a key determinant of NMDA receptor activation, these findings imply that NMDA-dependent synaptic plasticity cannot be described by a single timing rule referenced to the first somatic spike (Sjostrom et al., 2001). Instead, the outcome of NMDA-dependent plasticity depends jointly on the precise dendritic location of the synapse and the temporal structure of somatic spiking. For example, in proximal apical dendritic regions (e.g., near oblique branches), where distance-dependent attenuation is modest and bAP amplitudes remain relatively large (Stuart et al., 1997), synchrony with the first spike in a train is sufficient to ensure substantial dendritic depolarization (Figure 1B). In contrast, in distal apical compartments such as the apical nexus and tuft (Figure 1A), synchrony with later spikes in a high-frequency train may be required for an EPSP to coincide with a depolarized dendritic membrane (Figure 1C).

In summary, the observation that a single dendritic compartment (e.g., distal apical dendrite) can exhibit either bAP amplitude loss or gain, highlights that AP backpropagation efficacy is highly state-dependent and shaped by dendritic location, somatic firing frequency, and the timing of synaptic input relative to spike order. Consequently, NMDA-dependent plasticity cannot be reduced to a single “pairing window” referenced to the first spike. Rather, at least four interacting factors, (i) dendritic location, (ii) somatic firing frequency (Waters et al., 2003) their Figures 4, 5; Short et al., 2017 their Figures 1, 2), (iii) firing duration, and (iv) EPSP timing relative to spike order (1st, 2nd, 3rd, etc.), can strongly influence both the probability and magnitude of NMDA receptor activation.

### 2.3 Random bAP amplitude variations from a moment to moment basis

In distal apical branches of pyramidal neurons, bAP propagation often operates near the threshold for failure. Small differences in dendritic diameter, branching geometry, and the local balance of synaptic/neuromodulatory conductances can substantially alter invasion efficacy (Hoffman et al., 1997; Tsubokawa and Ross, 1997; Hoffman and Johnston, 1999; Golding et al., 2001). While modulation-dependent changes are expected, large trial-to-trial fluctuations can also occur without explicit experimental manipulation (Short et al., 2017). In that study, somatic current injections evoked APs while dendritic Ca^2+^ signals were monitored in distal apical tuft branches at regular intervals. In 37% of neurons (57/127), some trials could differ so markedly as to suggest genuine failures of bAP invasion in one or more tuft branches (Figure 1D, Failure). Short et al. (2017) termed this phenomenon “*AP flickering*,” reflecting alternation between successful invasions and failures across trials. Because each neuron was sampled repeatedly (10–33 trials), the authors assessed whether flickering followed a reproducible pattern within a given cell. Instead, the timing of strong versus weak AP-evoked Ca^2+^ signals appeared random and was not predictable from trial order. Thus, even in a fixed dendritic branch and in the absence of overt manipulation, distal tuft compartments can experience moment-to-moment variability in bAP efficacy; i.e., stochastic behavior of a bAP.

### 2.4 bAP amplitude differences at the same path distance from the cell body

Early work also demonstrated that dendritic branches at the same path distance from the soma can experience very different bAP-associated Ca^2+^ influx during the same somatic AP (Spruston et al., 1995, their Fig. 4A). During modest spike trains (~20 Hz), somatic AP amplitudes decline slightly with spike order, but dendritic attenuation is often much stronger. Because later spikes propagate less effectively into distal dendrites, branch points can become sites of selective propagation failure. If sister branches differ slightly in diameter, membrane conductance composition, or recent local synaptic activity, invasion may succeed in one branch (Figure 1E, loc. 1) while failing in the other (Figure 1E, loc. 2), producing large amplitude discrepancies despite equal path distance (Spruston et al., 1995; Zhou et al., 2015). These observations underscore that path distance alone does not determine dendritic depolarization during bAP signaling; branch identity and local state can be decisive for synapses attempting to engage in STDP. In contrast to these experimental evidence (Spruston et al., 1995; Zhou et al., 2015), most STDP studies rely primarily on path distance from the cell body (Sjostrom and Hausser, 2006), while neglecting the branch-specific and probabilistic nature of bAP invasion. Under identical conditions, some distal dendritic branches are invaded by bAPs (Figure 1E, loc. 1) whereas others are not (Figure 1E, loc. 2), rendering invasion outcomes difficult to predict and inherently stochastic.

### 2.5 bAP failures induced by successive branch points

As trains of bAPs propagate along the apical axis, they encounter successive branch points where the parent dendrite bifurcates into daughter branches (Figure 1F). Simultaneous optical recordings from multiple daughter branches reveal that the actual number of bAP-associated Ca^2+^ transients can differ markedly among branches of a different branching order; e.g., secondary vs. tertiary (Zhou et al., 2015). In one paradigm, three somatic APs (a triplet) are evoked while Ca^2+^ transients are imaged in several dendritic sites. In the Ca^2+^ imaging trace, three distinct Ca^2+^ peaks indicate successful invasion of all three spikes; however, more distal sites can exhibit only two, one, or zero peaks at the same time, consistent with progressive loss of later spikes at successive branch points (Figure 1F). For example, in location 1, Zhou et al. observed three Ca^2+^ transients (Figure 1F, loc. 1), whereas, more distal locations, loc. 2, loc. 3, and loc. 4 exhibited two, one, and zero APs, respectively (Figure 1F). Each successive branch point (BP-1 to BP-3) caused a reduction of one Ca^2+^ transients from the end of the train. As a triplet of bAPs progresses through the dendritic tree, at each new branch point there is one less bAP that successfully invaded the branch (Figure 1F). Later spikes are particularly vulnerable, plausibly due to cumulative Na^+^ channel inactivation that develops during repetitive firing (Migliore, 1996; Colbert et al., 1997; Jung et al., 1997), as well as branch-specific shunting inhibition or activation of K + conductances (Hoffman et al., 1997). The result is spatially heterogeneous bAP invasion, in which adjacent branches can experience quantitatively different number of successful dendritic spikes during the same somatic spike train (Spruston et al., 1995; Zhou et al., 2015; Short et al., 2017). Functionally, branch points may therefore act as dynamic “gates” that regulate how many spikes invade particular dendritic subtrees, potentially under control of synaptic and neuromodulatory inputs (Hoffman et al., 1997; Tsubokawa and Ross, 1997; Hoffman and Johnston, 1999; Larkum et al., 2001; Zhou and Antic, 2012).

Taken together, the five forms of amplitude variability summarized in Figure 1 indicate that, in many dendritic regions, the bAP is better described as a noisy, state-dependent signal rather than a fixed, deterministic waveform. Consequently, synaptic contacts on distal branches may not be informed of somatic spiking with complete reliability on every trial. Section 2 of this article therefore presents experimental evidence supporting the view that bAPs constitute a stochastic electrical signal, whereas in the following section (Section 3), we discuss the potential functional implications of this stochasticity.

## 3 Impact on information processing

### 3.1 Stochasticity of electrical signals

Stochasticity (often operationalized as “noise” and “trial-to-trial variability”) is not merely a nuisance in neural systems. In many regimes it is an essential feature that shapes how circuits encode uncertainty, explore alternative hypotheses, and compute under biophysical constraints. Several functional roles have been proposed for stochastic processes in brain information processing.

- Stochasticity as a constraint on encoding and reliability. At the biophysical level, randomness arises from ion-channel gating, synaptic release, and other microscopic processes. These sources propagate to variability in spike timing and population activity, setting limits on precision and motivating redundancy, averaging, and error-correction strategies (Faisal et al., 2008). We propose that variability in bAP amplitude in distal dendritic branches (Figure 1) contributes to this broader stochasticity in neural information processing, thereby increasing the reliance on averaging and error-correction mechanisms (Faisal et al., 2008).
- Probabilistic representation of uncertainty. Experimentally observed neural variability need not be interpreted as mere noise that corrupts sensory signals; instead, it may reflect a principled representation of uncertainty. In sampling-based and probabilistic theoretical approaches to neuronal computation, moment-to-moment neural fluctuations are informative, encoding posterior uncertainty rather than point estimates (single best estimates) (Orban et al., 2016). Under these frameworks, neural populations are assumed to represent not a single best estimate of a stimulus or latent variable, but an entire probability distribution; “*the posterior*.” This posterior distribution formalizes the brain’s updated belief about an uncertain or hidden variable, obtained by combining prior expectations with incoming sensory evidence. Intuitively, it can be understood as the brain’s internal probability map of what the external world might be, given both what it already expected and what it has just sensed.
- Decision-making and inference under uncertainty. Stochastic dynamics can support evidence-accumulation and Bayesian-like decision processes, especially when the system must integrate noisy sensory evidence over time. This is linked to probabilistic coding viewpoints, or probabilistic coding perspectives (Beck et al., 2008).
- Escaping local minima, flexible dynamics, and exploration. Noise can help networks avoid pathological stability (getting “stuck”) and promote transitions among metastable states, useful for exploration, switching, and flexible computation (Deco et al., 2011; McIntosh and Jirsa, 2019).
- Learning and generalization. Stochasticity can regularize learning (preventing overfitting), promote exploration in reinforcement learning-like settings, and shape synaptic updates when plasticity rules operate on both noisy presynaptic and noisy postsynaptic activity. While details depend on model class, some frameworks treat noise as beneficial for robust learning in variable environments (Faisal et al., 2008). Stochastic bAP signaling may belong to the broader class of “*noise-can-help*” mechanisms, in which variability enhances computational power in neural circuits (McDonnell and Ward, 2011).

### 3.2 Stochasticity of backpropagating APs and STDP

The potential impact of stochastic bAPs on synaptic plasticity, particularly STDP, is illustrated in Figure 2A. In canonical STDP, a dendritic EPSP interacts with a bAP at or near the synaptic contact, often in distal apical dendrites (e.g., apical tuft branches). Both (i) synaptic transmission and (ii) bAP invasion can be conceptualized as probabilistic “gates” that are not open on every trial (Figure 2A, Random Gate). In Figure 2A, the random gate for synaptic transmission is colored orange, while the gate for AP Backpropagation is colored green.

To illustrate the consequences of this stochasticity, consider a simple numerical example. The probability of successful neurotransmitter release at many neocortical synapses can be low (e.g., Pr ~ 0.2; Volgushev et al., 2004). Similarly, in a substantial subset of pyramidal neurons, somatic action potentials do not consistently produce robust Ca^2+^ signals in individual apical tuft branches, a phenomenon often referred to as AP “flickering” [observed in ~37% of cells; (Short et al., 2017)].

Suppose a neuron exhibits stochastic bAP propagation such that a given bAP successfully invades a particular dendritic branch on approximately half of trials (Pr = 0.5). If synaptic transmission and bAP invasion are independent processes, the probability that both gates (Figure 2A, orange and green) are open during a given pre-post pairing is given by the product of their individual probabilities (0.2 × 0.5 = 0.1). Thus, even when both presynaptic and postsynaptic neurons fire action potentials, only one out of 10 pairing events would be expected to produce an effective EPSP-bAP interaction capable of driving STDP. This simple thought experiment highlights that, particularly at distal dendritic synapses, plasticity induction may be inherently probabilistic, emerging from the coincidence of multiple stochastic cellular events rather than from deterministic spike timing alone.

These probabilistic gates are not unique to synaptic release and AP backpropagation. Many forms of electrical signaling in pyramidal neurons operate with state-dependent success probabilities or fluctuating values (Figure 2B). Importantly, these probabilities are not fixed; they can be modulated by excitation, inhibition, and neuromodulators (e.g., glutamate, GABA, acetylcholine, norepinephrine), as well as by spike frequency and recent activity history (Figure 2B, Probability Modulation).

To illustrate a potential computational consequence, consider a simplified decision scenario in which two similarly sized neural assemblies compete to represent alternative interpretations of sensory input. If electrical signaling were perfectly reliable everywhere (the gates are always open, Pr = 1.0), both assemblies could be driven strongly, reducing contrast and making discrimination difficult (Figure 2C). Introducing probabilistic signaling at multiple stages (Figure 2A, Random Gates, Pr < 1.0) can amplify small differences in inputs into larger differences in population output, increasing separability of competing representations (Figure 2D). In this thought experiment, *Network 1* codes “red apples” and *Network 2* becomes active when “green watermelons” appear in the sensory field. Strong activation of Network 1 would bias the brain’s percept toward red apples, whereas strong activation of Network 2 would bias it toward green watermelons. If electrical signals propagate deterministically (success rate of 100%, Pr = 1.0), both networks will recruit many neurons and produce similar average activity, undermining a clear decision (Figure 2C). If, instead, each signaling step incorporates state-dependent stochasticity (random gating with Pr < 1.0), then modest differences in: (i) sensory drive, (ii) neuromodulatory tone, or (iii) brain state can be amplified into larger differences in population-averaged responses, yielding a clearer winning network (Figure 2D, Network 1 emerges as the winner), ultimately giving rise to the conscious perception of a red apple.

To summarize, neural information processing is largely analog and distributed across neuronal populations, whose activity patterns compete for dominance; the winning ensemble determines the resulting percept or memory representation (Desimone and Duncan, 1995; Reynolds and Chelazzi, 2004; Dehaene and Changeux, 2011). In other words, neural ensembles compete, and the winning population determines the resulting perceptual or cognitive representation (Figure 2D).

## 4 Concluding remarks

In a substantial subset of cortical and hippocampal pyramidal neurons, bAP invasion into apical tuft branches (distal dendrites) exhibits trial-to-trial variability consistent with stochastic propagation (Figure 1). Such variability should not be viewed solely as a defect: it can impose limits on precision, motivate redundancy, averaging, and potentially support evidence accumulation, and probabilistic computation in cortical circuits.

Apical Trunk Void of Dendritic Spines. In contrast to distal dendrites, bAP propagation into proximal dendrites is often effectively deterministic: each somatic spike reliably depolarizes proximal compartments (Pr = 1.0). Interestingly, the density of synaptic contacts is relatively low in the proximal apical trunk (Figure 2A, Apical Dendrite), where bAPs are most reliable, whereas distal compartments, including tuft branches (Figure 2A, Apical Tuft Branch), where bAPs are not reliable, are densely populated with dendritic spines, i.e., synaptic contacts (Garcia-Lopez et al., 2006; Szots et al., 2026). Consistent with this dendritic spine gradient, thick-tufted layer 5 pyramidal neurons receive substantial excitatory input from supragranular layers, particularly from layer 1 (Larkman, 1991), where the apical tuft resides, and where bAPs display pronounced spatiotemporal variability (Figure 1). Dendritic spines increase the membrane surface area of dendrites and therefore influence backpropagating action potentials (bAPs) primarily through their effects on passive electrical properties: membrane capacitance (Cm) increases, and membrane resistance (Rm) decreases (Wilson, 1984; Koch and Zador, 1993). The resulting increase in capacitive load requires more current to depolarize the dendritic membrane, which can broaden and slow bAP waveforms in spine-rich dendritic regions (Segev and London, 2000). In distal apical dendrites, the additional load imposed by spines can contribute to strong attenuation, or even failure, of bAP propagation unless supported by sufficient Na^+^ conductances on dendritic spines (Tsay and Yuste, 2002). The strategic placement of synapses in distal dendritic regions (where bAP invasion spans a wide dynamic range, from strong depolarization ~60 mV, to near-complete failure <20 mV), may be computationally advantageous. Such distal positioning of synaptic contacts enables branch-specific gating (Figure 2A, green gate) and introduces greater dependence on: (i) redundancy, (ii) averaging, (iii) evidence accumulation, and (iv) error-correction strategies. In this view, stochastic variability in bAP signaling is not merely incidental but may represent a functional feature that supports flexible, state-dependent cortical processing.
